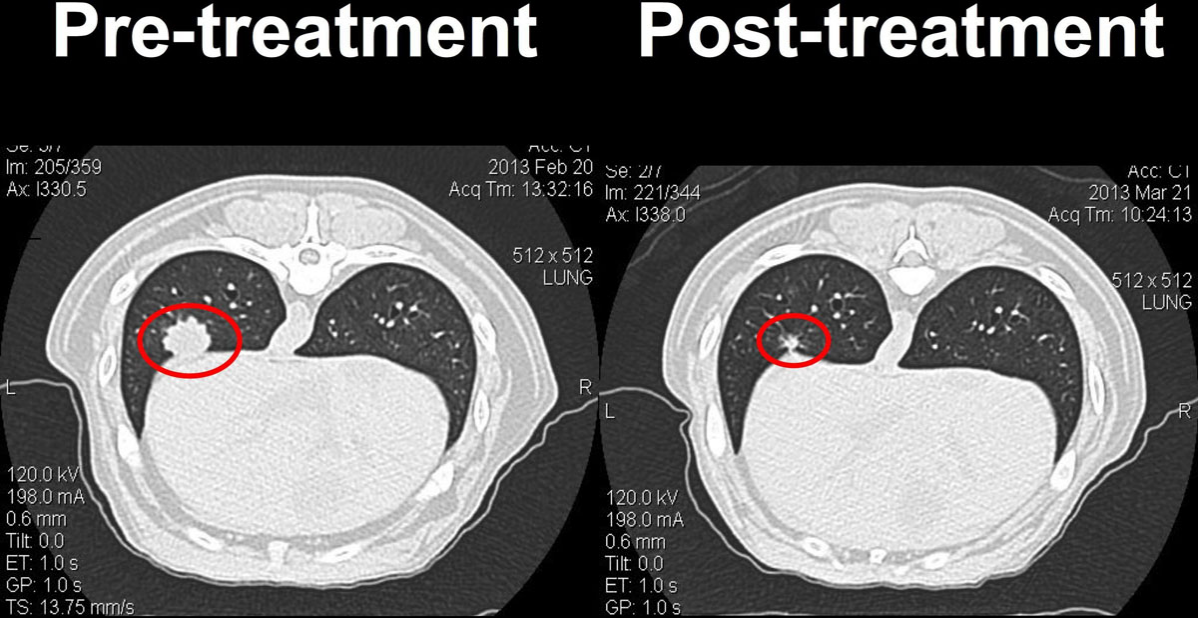# Combined radiotherapy and immunotherapy using CPG oligodeoxynucleotides and indolamine 2,3 dioxygenase (IDO) blockade

**DOI:** 10.1186/2051-1426-1-S1-P256

**Published:** 2013-11-07

**Authors:** Arta M Monjazeb, Steven K Grossenbacher, Gail D Sckisel, Robert Canter, Ellen E Sparger, William Culp, Michael S Kent, William J Murphy

**Affiliations:** 1 Radiation Oncology, UC Davis Health System, Sacramento, CA, USA; 2 Dermatology, UC Davis Health System, Sacramento, CA, USA; 3 Surgery, UC Davis Health System, Sacramento, CA, USA; 4 Veterinary Medicine, UC Davis Health System, Davis, CA, USA

## Background

Pre-clinical and clinical data demonstrate the effectiveness of combining RT and CpG immunotherapy. There is data to suggest that the effectiveness of CpG immunotherapy may be limited by induction of the immunosuppressive enzyme, IDO. We test the efficacy and safety of combining RT and CpG with blockade of the immunosuppressive enzyme, IDO, using 1-Methyl D-Tryptophan (1-MT).

## Methods

Tumor bearing mice were treated with RT + IT. IT consisted of intratumoral CpG and 1-MT administered in the drinking water. Tumor growth, survival, toxicity and the immune profile of various tissues were assessed. Based on preliminary results a similar clinical trial was initiated at the UC Davis Veterinary Cancer Center testing this approach for spontaneous metastatic cancers in companion canines.

## Results

In mice therapy improved overall survival, decreased lung metastases, decreased mean tumor growth, increased percentage of activated dendritic cells in tumor draining lymph nodes, and decreased levels of regulatory T-cells. No treatment related toxicities were observed.

In the canine clinical trial 6 patients have been enrolled to date. No treatment related toxicities have been observed. Preliminary results indicate a robust response at the primary treated disease site but also indicate the induction of systemic anti-tumor immunity as demonstrated by regression or stability of un-irradiated metastatic disease. The treatment has drastically reduced the level of regulatory T-cells in the tumor, draining lymph nodes, and peripheral blood in some animals.

## Conclusions

Combining RT and IT is more effective than either therapy alone. Preliminary results in spontaneous canine cancers also show promise. Further study is needed to determine the immunologic mechanism of this therapy. This therapy has limited toxicity in both mice and canines and is being considered for translation to human studies.

**Figure 1:**